# The Utilization of Bee Products as a Holistic Approach to Managing Polycystic Ovarian Syndrome-Related Infertility

**DOI:** 10.3390/nu15051165

**Published:** 2023-02-25

**Authors:** Ahmad Ali, Additiya Paramanya, Payal Poojari, Damla Arslan-Acaroz, Ulas Acaroz, Aleksandar Ž. Kostić

**Affiliations:** 1 Department of Life Sciences, University of Mumbai, Vidyanagari, Mumbai 400098, India; 2 Department of Biochemistry, Faculty of Veterinary Medicine, Afyon Kocatepe University, 03200 Afyonkarahisar, Turkey; 3 ACR Bio Food and Biochemistry Research and Development, 03200 Afyonkarahisar, Turkey; 4 Department of Food Hygiene and Technology, Faculty of Veterinary Medicine, Afyon Kocatepe University, 03200 Afyonkarahisar, Turkey; 5 Department of Food Hygiene and Technology, Faculty of Veterinary Medicine, Kyrgyz-Turkish Manas University, Bishkek KG-720038, Kyrgyzstan; 6 Department of Chemistry and Biochemistry, Faculty of Agriculture, University of Belgrade, Nemanjina 6, 11080 Belgrade, Serbia

**Keywords:** bee products, polycystic ovarian syndrome (PCOS), systematic review

## Abstract

Bee products, including honey, have been utilized since ancient times for nutritional and therapeutic purposes. Recently, other bee products such as bee pollen, royal jelly, and propolis have caught a lot of attention. Being high in antioxidants and bioactive compounds, these products have established their applications in the pharmaceutical field as supplementary or alternative medicines. This review focuses on their use against polycystic ovarian syndrome (PCOS)-related infertility. A systematic search of electronic databases including PubMed, Web of Science ScienceDirect, and Google Scholar was conducted from their inceptions up to November 2022. Studies with a small sample size, studies with inconclusive data, and pre-prints have been excluded. A narrative synthesis was performed during draft preparation after the authors independently performed a literature search. A total of 47 studies were finalized for the review. It can be observed that in vivo data on the use of bee products in treating PCOS mostly deals with their use in synergism with the PCOS medicines to enhance their effect and/or curb their side effects; however, clinical trials for the same are limited. With the amount of data being limited, it is difficult to map out the mechanism by which these products act in managing PCOS inside the human body. The review gives detailed insights into the reversal and restorative properties of bee products against the aberrations in reproductive health caused by PCOS.

## 1. Introduction

**Figure 1:**
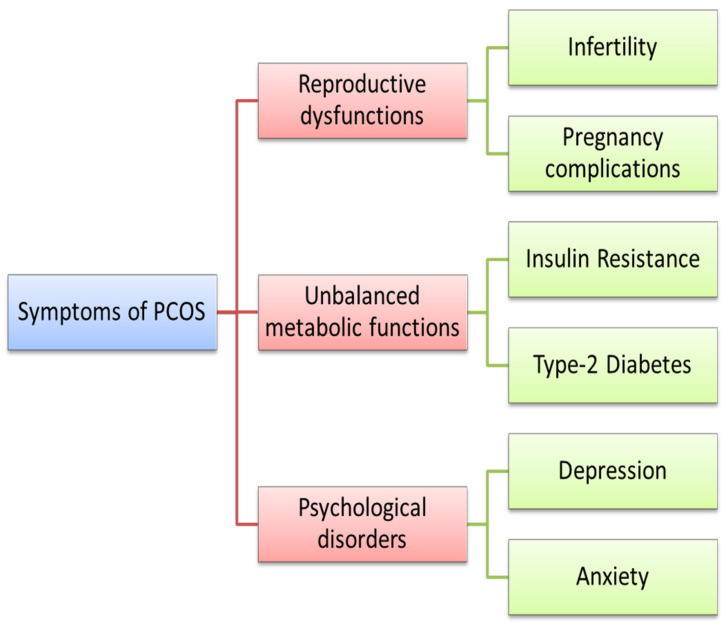
Major symptoms associated with polycystic ovarian syndrome (PCOS).

John Hopkins Medicine describes polycystic ovarian syndrome (PCOS) as a hormonal disorder in which the ovaries produce an irregular quantity of androgen. As the name suggests, the syndrome wherein numerous cysts are observed in the ovaries of women in their reproductive ages. It is one of the most common endocrinological and metabolic disorders affecting 8–13% of women [1]. However, it is assumed that most patients go undiagnosed as the symptoms may be generalized or associated with other disorders (Figure 1). There is no treatment or cure for PCOS per se, but the majority of medications deal with the relief of a wide array of symptoms associated with the disorder [2]. This review tries to study and summarize the available information on the use of bee products as a holistic approach to PCOS-related infertility.

A branch of alternative medicine that involves the application of bee products made by honeybees involving honey, bee pollen, royal jelly, bee venom, and propolis is known as Apitherapy. Apitherapy uses these bee products to treat various human illnesses and, in some cases, also for treating wounds. Various studies and experiments are now being conducted to understand the effectiveness of the healing properties of bee products in tackling different diseases. These products are primarily used to manage certain non-curable diseases such as neurological disorders, immunological conditions, certain types of allergies, and hormonal regulation. Traditionally, bee products are used in many cultures and ethnicities as a therapeutic agent in curing many aids. These products are rich in various biological components and activities beneficial for human health [3].

Bee products, especially honey, have been used since ancient times as a safer alternative to sugar. Being rich in antioxidants, honey indirectly helps PCOS patients by lowering the risk of oxidative stress, inflammation, and type-2 diabetes [4]. The second bee product selected for the review was honeybee venom. Responsible for a bee sting, it is a poisonous product containing a wide array of complex blends of peptides, amines, etc. Its anti-inflammatory property has been widely studied; thus, it seemed it had potential therapeutic properties in PCOS. Royal jelly possesses minimal toxicity after honey amongst the other bee products and has recently caught a lot of attention [5]. The diversity of bioactive compounds present in royal jelly helps in soothing various symptoms of PCOS [3]. Propolis, a waxy substance produced by honeybees, was the final compound selected owing to its antioxidant properties. This systematic review is a limited qualitative narrative of the available data for the literature on PCOS and the use of bee products such as honey, honeybee venom, royal jelly, propolis, and bee pollen in its treatment. The data taken into consideration here are thorough clinical investigations mostly dealing with rat models that use bee products as a holistic approach and/or their supplementation with other medicines. 

## 2. Study Design and Research

The literature study used databases including PubMed, ScienceDirect, Google Scholar, and Web of Science. A systematic search of the data up to November 2022 was performed. The search was a combination of clinical trials, case reports, meta-analysis, review, and systematic review. Search terms included Polycystic Ovarian Syndrome or PCOS and bee products, honey, honey bee venom, royal jelly, and bee pollen. The search was also expanded by referring to relevant citations in the selected literature [6]. Meta-analysis could not be performed as most of the literature available was qualitative. Moreover, there was a lot of variation in the method of studies and their outcomes. 

**Figure 2:**
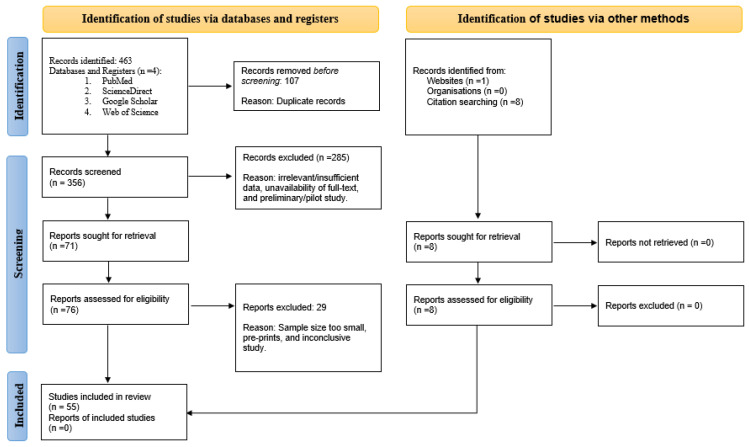
PRISMA (Preferred Reporting Items for Systematic Reviews and Meta-Analyses) flow diagram for data extraction.

A narrative synthesis was employed among the authors to finalize the literature to be utilized in formulating the review. Authors A.P. and P.P. independently performed a data search and compared their research before drafting the manuscript. This was decided based on the utilization of bee products as a holistic approach and/or alternative or supplementary medicine for the relief of PCOS symptoms, especially infertility. Duplicated data were identified using EndNote 20.2.1 by Clarivate. The various steps in the systematic review have been depicted using a PRISMA 2020 flow diagram (Figure 2). Additionally, the relevant studies related to the effects of bee products on PCOS are summarized in Table 1.

## 3. Honey

**Figure 3:**
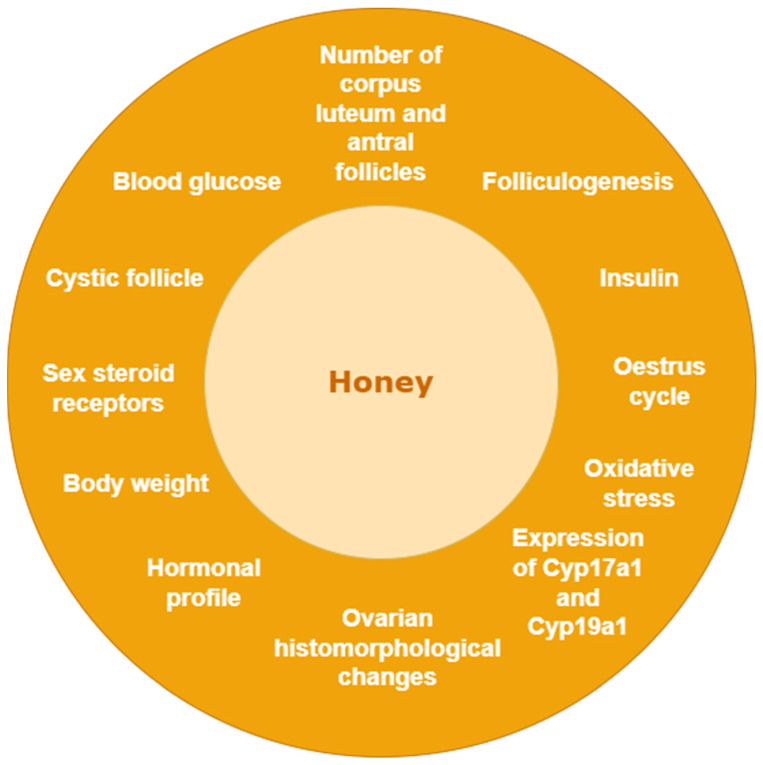
Honey-affected parameters related to PCOS.

Ancient literature mentions honey as a product used for its nutritional and healing properties. Honey comprises a supersaturated solution of carbohydrates (90–95% of dry mass) mixed with approximately 17% of water [20]. The characteristics of honey vary depending on the floral source, type of bee, environmental conditions, and/or extraction, processing, and storage conditions of packed honey [21]. Moniruzzman and colleagues identified more than 400 volatile and semivolatile compounds in honey which include C13-norisoprenoids, benzyne derivatives, monoterpenes, sesquiterpenes, fatty acids, alcohols, aldehydes, esters, and a wide array of organic acids such as aspartate, acetate, alpha-ketoglutarate, shikimate, etc. [22,23]. Although honey has been proven to have innumerable therapeutic properties, direct evidence of the use of honey in PCOS treatment is limited. Honey affected parameters related to PCOS were presented in Figure 3.

A group of researchers used a traditional prescription of Kyung-Ok-Ko (KOK) in dehydroepiandrosterone (DHEA)-induced PCOS rat model to determine its suitability as a therapeutic and preventive natural alternative for suppressing PCOS. KOK contains a decoction of six ingredients, namely, *Aquillaria agallocha* Roxburgh, honey, *Lycium Chinese* Mille, *Panax ginseng* C.A. Meyer, *Poria cocos*, and *Rehmannia glutinosa* Liboschitz var. purpurae Makino. Preadministration of KOK in DHEA-injected rats showed mitigation of increased mRNA levels of tumor necrosis factor-α, interleukin-1β, 6 and 8, and inducible nitric oxide synthase (iNOS) in rat ovaries with PCOS. A considerable decrease in the number of follicular cysts, the weight of the body and ovary, serum glucose, and estradiol levels were also observed in such rats [24]. 

Letrozole as a nonsteroidal aromatase inhibitor reduces the extent of estrogen released by the body and is widely used for inducing PCOS-like symptoms in rats. Letrozole-induced PCOS causes downregulation of leukemia inhibitory factor (LIF), bone morphogenetic protein (BMP), and the Kit Ligand (KITL) [25]. Kelulut honey (KH) almost did not affect LIF expression; however, it could significantly reverse KITL and BMP-1 downregulation [7]. An increase in ovulation rate causes an increased number of corpus luteum, and the presence of antral follicles indicates the occurrence of folliculogenesis. The research also showed the histological beneficial effects of KH wherein the anomaly caused by letrozole was reversed by KH used in combination with metformin and clomiphene, and the treated PCOS rats showed high counts of corpus luteum and antral follicles, while cystic follicles number had decreased considerably [8].

In rodent ovaries, upregulation of gene expression of Cyp17a1 protein is observed in theca cells of large antral and preovulatory follicles [26]. KH has been reported to decrease the Luteinizing hormone (LH) hypersecretion which in turn reduces Cyp17a1 overexpression [10]. Tualang honey was found to mediate an antidepressant effect on stressed ovariectomized rats. The mechanism for this is unknown; however, the researchers believe the outcome could be possibly by reversal of anomalies in the hypothalamic–pituitary–adrenal axis or by elevation in the brain-derived neurotrophic factor concentration [27] Aberrant expression of Estrogen receptors alpha (ERα) and beta (ERβ) causes folliculogenesis and ovulatory dysfunction in PCOS patients [28]. Kamal and colleagues found out that KH-fed rats showed significant restoration of mRNA expression of both ERα and Erβ, while another study reports downregulation of ER expression by Taulang and Manuka honey [9,29]. 

No direct evidence of the use of honey in the treatment of PCOS or its symptom relief has been reported in human trials. Most herbal medicines used in PCOS use honey as a demulcent [21].

## 4. Honeybee Venom 

**Figure 4:**
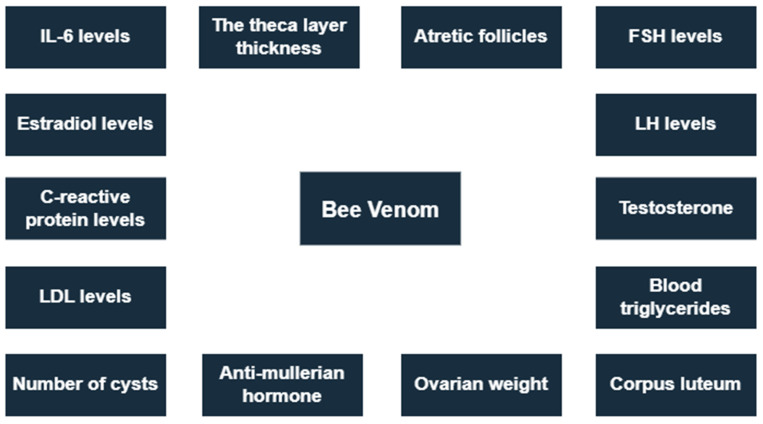
Bee venom affected parameters related to PCOS.

Bee venom (BV) is poison released by female worker bees in the hive that is responsible for bee stings. Just like other venoms produced by different animals, bee venom is also studied for its therapeutic properties. Some of the most commonly known active constituents of bee venom are melittin, adolapin, apamin, and MCD-peptide—a complex blend of peptides. Bee venom is also rich in enzymes such as PLA2 and bioactive amines (e.g., epinephrine and histamine), and minerals. The established components of bee venom are studied for various human diseases, namely, amyotrophic lateral sclerosis, neurodegenerative diseases such as Alzheimer’s and Parkinson’s diseases, and different classes of cancers. Bee venom has also shown extraordinary antiviral activity against human immunodeficiency (HIV). Recently, the effects of bee venom have also been studied in the neuroendocrine segment for disrupted hormonal secretion affecting fertility in women [3]. Bee venom affected parameters related to PCOS were presented in Figure 4.

Studies conducted showed the effective modulatory effect of honey bee venom on hyperglycemia with regard to hyperandrogenism in PCOS-induced Wistar rats. Additionally, a decreased hormonal level of Testosterone and Estradiol along with decreased blood sugar levels was observed in HBV-treated rats. Some noteworthy morphological changes were also noted in the reproductive organ in HBV-treated rats in comparison to the PCOS group. The thickness of the theca layer, the number, as well as the diameter of the cyst developed in the ovary were significantly decreased. Moreover, increased corpus luteum, a healthy sign of ovulation, was observed in HBV-treated ovaries. The beneficial effect of HBV against PCOS may be due to the inhibitory effect of HBV on TNF𝛼 level [19].

Another study showed similar morphological results in PCOS-induced Wistar rats. An attempt to study the inflammatory effect of HBV on Wistar rats induced PCOS by estradiol showed an important increase in the level of C-reactive protein (CRP) in the HBV-treated group with PCOS. The immunohistochemical analysis showed a decrease in the expression of the cyclooxygenase-2 (COX-2) enzyme, decreasing the inflammatory stimuli in PCOS showing positive effectiveness of HBV in the PCOS group. This positive effect of HBV may be caused by the inhibitory effect of HBV on COX-2 and CRP levels [30].

A similar study conducted to determine the association of CRP with follicle health represented related results showing a decrease in the CRP levels was associated with improved follicle health due to the anti-inflammatory effect of HBV (0.5 mg/kg—for 14 days) on the PCOS group. The decrease in the CRP level also reduces the metabolic features and its associated disorder in PCOS [18].

Numerous other studies also represented a positive anti-inflammatory effect of HBV as a therapeutic agent suppressing the levels of major inflammatory mediators such as vascular endothelial growth factor (VEGF), COX-2, and interleukin-6 (IL-6) in PCOS-induced Wistar rats by estradiol valerate [16].

COX-2 involvement is observed during inflammation and extensive cell proliferation which is influenced by prostaglandin synthesis. COX-2 in PCOS has a major proliferative effect on the theca layer of the ovary where the process of ovulation takes place. The overexpression of COX-2 is through TNF𝛼 affecting the thickness of the theca layer of the ovary [31].

Attempts to study the effect of HBV on Anti-Mullerian Hormone (AMH) and lipid profile. Decreased levels of blood triglycerides and low-density lipids were observed in the HBV-treated PCOS group of rats. Similar results were observed in the case of AMH when treated with HBV. The benefits of HBV were successfully observed in the suppression of AMH factor and significant suppression of LDL and triglycerides levels [17]. The reduction in lipid levels in PCOS can regulate the hyperandrogenism in the disorder. 

One of the most innovative studies for managing PCOS involves a laparoscopic bilateral intraovarian injection of bee venom. The 15 subjects of PCOS, resistant to medical treatment, were injected with 0.1 mL of BV into the ovarian stroma. The results of the study indicated a reduction in LH, androstenedione, and testosterone in the subjects, promoting ovulation by 70%, and the pregnancy rate was 50% in those who showed signs of ovulation with no complication [32].

PCOS exhibits a variety of symptoms which can be categorized based on physical, psychological, metabolic, and neuroendocrine leading to reproductive dysfunction. These symptoms often lead to the manifestation of each other. Bee venom in general might be toxic in nature, and hence, its consumption for treatment of PCOS does not seem feasible. In a study of 46 PCOS patients divided into two groups who were on a high-low-calorie diet, participants were subjected to bee venom phonophoresis. Phonophoresis is a non-invasive technique that implies use of ultrasound waves to increase the transdermal drug delivery process. After 7 and 14 weeks of the treatment, a significant decrease in LH and LH/FSH ratio was observed. The treatment proving BV phonophoresis with guided physical activity and proper diet has a positive effect in managing obese PCOS women [15]. This is one of the exclusive examples of the use of a bee product wherein a direct indication of the effectiveness of bee venom on the treatment of PCOS is available.

## 5. Royal Jelly

**Figure 5:**
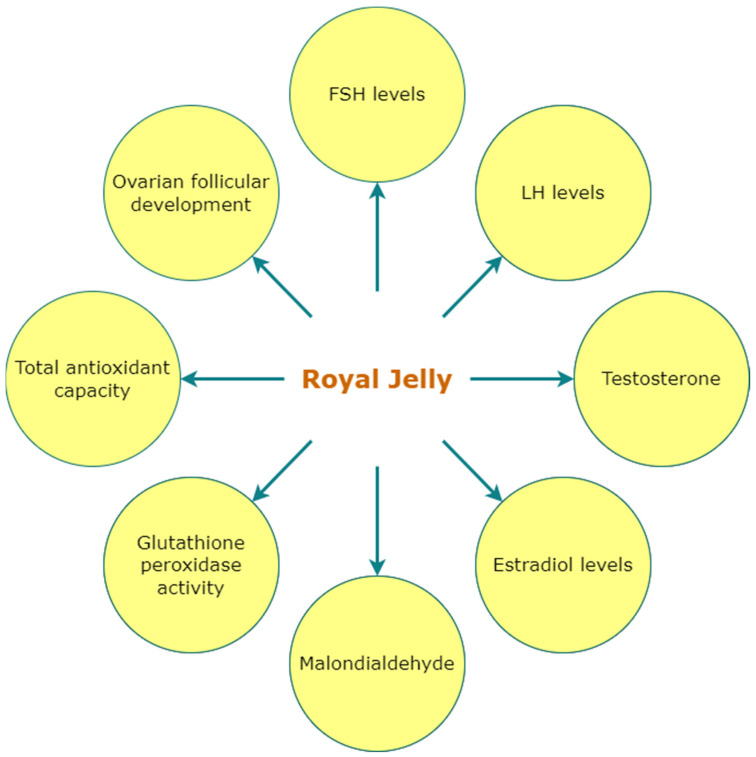
Royal jelly affected parameters related to PCOS.

Secreted by the hypopharynx of nurse bees, royal jelly is a honeybee secretion that is primarily used to meet the nutritional needs of growing larvae and adult queens. While developing a new queen bee, the workers construct special queen cells. The larvae in the specialized cells are constantly fed with an abundant amount of royal jelly to facilitate the development of mature queen morphology for fully grown and developed ovaries required to lay many eggs. The chemical composition of royal jelly is believed to favor such a type of development in queen bees. An amount of 60–70% of the royal jelly is composed of water, different forms of sugar, dicarboxylic acid, and a rare and unique type of short hydroxy fatty acid with 8–12 C atoms [33]. Most important fatty acids found in royal jelly are sebacic acid (SA), 10-hydroxy-2-decanoic acid (10-H2DA), and 10-hydroxydecanoic acid (10-HDA) [34]. A wide variety of proteins is dominant ingredients of royal jelly that are composed of nine major proteins of 49–87 kDa. Phosphorylation and glycosylation of these proteins are crucial for some vital biological processes involving glycoproteins, such as immunity, cell adhesion, differentiation, and growth. These major proteins of royal jelly regulate the development of female larvae and contribute to cell proliferation, cytokine suppression, etc. [35]. Apart from this major component, royal jelly also constitutes a diverse variety of phenolic compounds, free amino acids, carbohydrates, biogenic elements, vitamins, and some bioactive substances [36]. Royal jelly affected parameters related to PCOS were presented in Figure 5.

Royal jelly shows a significant effect in promoting ovarian follicle growth in immature rats. A study showed that a significant increment in ovarian and uterine weights also increased the serum levels of estradiol and progesterone. Along with the hormonal increase, a substantial enhancement in the quantity of corpora lutea and mature follicles was observed. The rats divided into four groups were subjected to 100, 200, and 400 mg/kg/day doses of royal jelly and showed improved folliculogenesis and an increase in ovarian hormones [37]

In another experiment, 40 immature rats were injected with testosterone propionate to induce hyperandrogenism and were randomly divided into 5 groups as control C, testosterone T, T + 100RJ (100 mg/kg/day of royal jelly), T + 200RJ (200 mg/kg/day of royal jelly), and T + 400RJ (400 mg/kg/day of royal jelly). The results reflected that the T + 200RJ group had a significantly increased FSH levels, notably lower LH, testosterone, and estradiol level in comparison to the T group in the experiment. The T + 200RJ group also showed a notable recovery from various stages of ovarian follicular development. Therefore, consumption of royal jelly at 200 mg/kg/day for 4 days demonstrated improved PCOS parameters [14].

In a similar experiment with 42 PCOS Wistar rats, were divided into six groups subjected to 100, and 200 mg/kg for 21 days. The PCOS + RJ group exhibited increased ferric-reducing antioxidant power and progesterone level, and a decreased level of nitric oxide and estradiol was also observed. Along with some hormonal improvement, the number of mature follicles and corpus luteum also enhanced significantly [38].

All the research listed under the use of royal jelly is animal trial. Detailed Human trial data were unavailable and/or inconclusive and, thus, have not been included in the review.

## 6. Bee Pollen 

**Figure 6:**
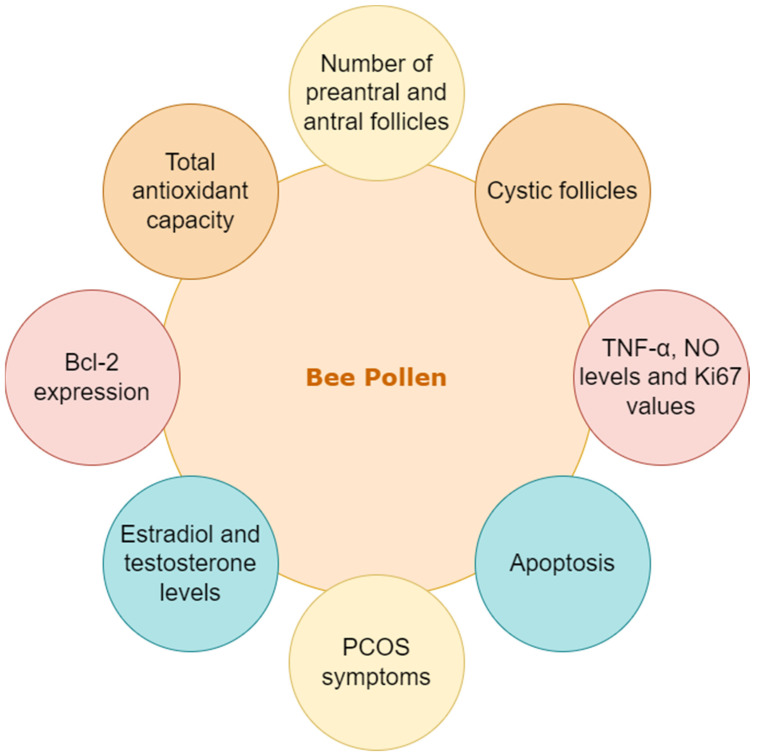
Bee pollen affected parameters related to PCOS.

Bee pollen is raw material collected by the worker bee to produce bee bread and ambrosia. This is the flower pollen collected by the worker bee and it is also a primary source of food in the hive. Bee pollen consists of varied plant-based compounds with expressed bioactivities [39]. It is also recognized as an excellent functional food ingredient [40]. Basic chemical substances such as important amino acids, proteins, carbohydrates and sugars (mainly fructose and glucose), lipids (such as linoleic, γ-linoleic, archaic, and Phospholipids) and fatty acids, and phenolic compounds (including catechins, flavonoids, phenolic acids, and leukotrienes) are presented in bee pollen [41]. Among flavonoids, there are mainly quercetin, isorhamnetin, kaempferol, and their derivatives, while in the group of phenolic acids, there is mainly chlorogenic acid), coenzymes, and enzymes as well as vitamins and bioactives (such as provitamin A and vitamins B1, B2, B6, C, D, and E, and acids: folic, pantothenic and nicotinic, and rutin, inositol, and biotin) [41,42,43]. Bee pollen affected parameters related to PCOS were presented in Figure 6.

A study designed to understand the effect of metformin and bee pollen on the levels of testosterone (T), estradiol (E2), total antioxidant capacity, and other apoptotic markers showed a similar effect of metformin and bee pollen in improving the levels of T, E2, and TAC and enhanced expression of apoptotic genes was performed [13]. In the case of the untreated PCOS group, E2 and T levels and Bcl-2 expression were enhanced. However, the expression of Cas-3, Bax, and Sirt1 was significantly decreased. In comparison to the treated PCOS group, there was a notable decrease in T and E2 including the expression of Bcl-2. Contrastingly, TAC and the expression of Cas-2, Bac, and Sirt1 were enhanced in the case of bee pollen and MET-treated groups [13].

A similar study aimed towards understanding the effect of metformin and bee pollen together and individually on Wistar rats. These rats were given bee pollen with 50, 100, and 200 mg/kg and metformin with 300 mg/kg either alone or in combination. On evaluating the results, the PCOS group treated with bee pollen and metformin showed increased levels of preantral, antral follicles, and cystic follicles were significantly reduced. The levels of NO and TNF𝛼 as well as the expression of Ki67 were suppressed. The study concluded that bee pollen when used synergistically or individually with metformin considerably improved the symptoms of PCOS [12].

The research listed here under bee pollen talks about significant differences in gene expression levels between control and bee pollen-supplemented rat models. Both rat models confirm the increase in the effectiveness of metformin in treating PCOS when supplemented with bee pollen confirming its synergistic effect [12,13]. 

## 7. Propolis 

**Figure 7:**
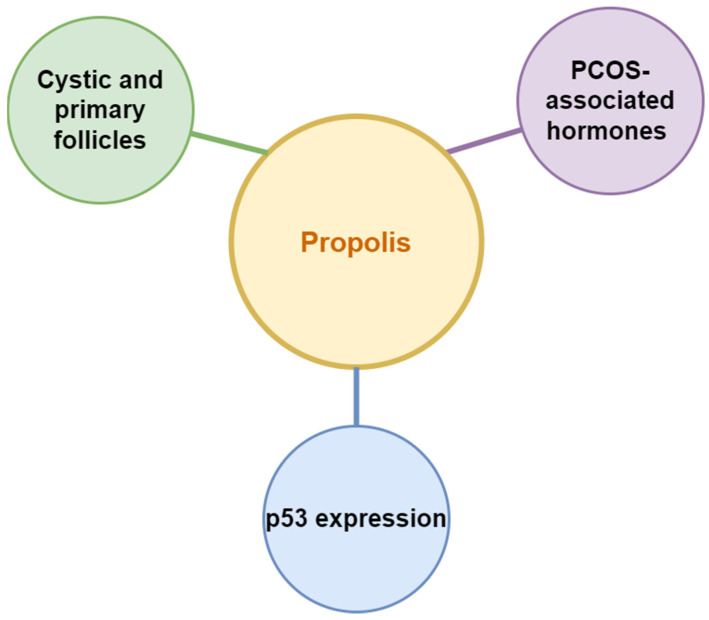
Propolis affected parameters related to PCOS.

Propolis is a waxy material produced by honeybees by collecting plant substances from different parts including exudates and buds. Generally, bees use propolis as a protective and constructive substance to protect the hives from any external invaders and extreme environmental conditions and repair their hives, respectively. However, propolis is known to have many beneficial effects on human health. Propolis is composed of waxes, resin, pollen, essential oils, and other organic compounds [44,45]. Chemical compounds such as: phenolics; benzoic acids and derivatives; cinnamic acid and its derivatives and cinnamic alcohol; triterpene and sesquiterpene hydrocarbons; benzaldehyde derivatives; other acids and respective derivatives; ketones, alcohols, and heteroaromatic compounds; terpene and sesquiterpene alcohols and their derivatives; aliphatic hydrocarbons; biogenic elements; steroid hydrocarbons and sterols; amino acids; and sugars are presented in propolis [46,47]. Propolis is known for its antioxidant, antibacterial, antifungal, antitumoral, and antidiabetic activities, and along with these activities, it has also shown a prominent result in allergies, rhinitis, and asthma [47]. Propolis-affected parameters related to PCOS are presented in Figure 7.

A study was conducted to understand the effect of propolis in PCOS rats and examined the effect of 50 and 150 mg/kg of propolis on p53 expression, ovarian folliculogenesis, serum progesterone, and LH level in PCOS rats. Experimental animals were divided into four different groups and were induced with letrozole for 21 days. The group which received propolis after 21 days of administration for 10 days showed a significant decrease in the number of cystic follicles comparing to the untreated group with a significantly higher level of LH in the propolis-treated group. However, there was no significant difference seen in the circulating P levels. Additionally, p53 immunoreactivity was not seen in any group. The study concluded that low concentrations of propolis cannot entirely improve the hormonal profile and the expression of p53 in PCOS; however, these concentrations can successfully control the ovarian follicular cell structure [11]. No human trial data are available on the use of propolis for PCOS management/treatment.

## 8. Potential Limitations

Honey is considered to be potentially safe for humans. However, honey is found to be frequently contaminated with yeasts, bacterial spores, and fungi [48]. Bee venom released during a bee sting causes systemic toxic reactions or allergic reactions in the first few minutes, which may further progress to anaphylactic shock. Such hypersensitive reactions on patients should be accounted for before suggesting the use of bee venom in PCOS treatment [49]. Similar hypersensitive reactions were observed in few patients on the first dose of royal jelly [50,51]. Bee pollen may cause allergic reactions including anaphylaxis on first consumption [52,53]. However, the more concerning part for bee pollen is the presence of contaminants such as pesticides, herbicides, heavy metals, toxins, etc., as reported in several studies from the literature [54,55]. A higher concentration (300 μg/mL) can activate mast cells and release inflammatory mediators resulting in allergy on consumption and application, and contact dermatitis on application [55].

Several studies have reported the limitations of usage of bee products in PCOS-related infertility. Different types of honey were found to have different effects on sex hormone signaling, wherein KH restored the mRNA expression of Erα and Erβ. On the other hand, Tualang and Manuka honey caused downregulation of expression of same transcripts in the study group. Several in vivo studies have shown PCOS-like symptoms in the rats using levastrol and DHEA. However, clinical trial reports are limited for similar kind of studies in humans. Similarly, a few studies showed histological evidence in human and rat models, most of these studies lacked the mechanism of the bee products in PCOS treatment. The data was also severely limited indicating that not much research has yet been focused on bee products as a holistic approach.

## 9. Concluding Remarks

There are major challenges in the management of PCOS. The present systematic review tries to collectively present all the data available for use of bee products in PCOS. Although Apitherapy has been used in traditional times for therapeutic and nutritional purposes, it has not been much researched for its pharmaceutical properties. Of all the bee products, honey, bee venom, bee pollen, and propolis have been tested in vivo for their use in PCOS. It is noteworthy that almost all the data available suggest the use of these bee products in synergism with the prescribed PCOS medicines, precisely, clomiphene and metformin.

Conclusively, the collected data reveals the potential for bee products to be of therapeutic potential in the relief of several symptoms of PCOS. These products work best in synergism with the available medications; however, in a few studies, it can be observed that the product alone could work effectively. Although the mechanism could not be determined, the high-antioxidant properties and presence of several bioactive compounds in bee products can be assumed to have contributed to the significant protection properties of bee products on PCOS. Moreover, the current review can provide insight into the gaps present in the literature for the use of bee products in PCOS and provide data required to conduct further studies on the same.

## Figures and Tables

**Table 1:** Summary of studies carried on the use of bee products in treatment and/or management of PCOS.

	Bee Product	Studied Parameters	POCS Induced by	Study Model	Outcomes of the Study	Author
1	Kelulut Honey 0.5, 1, and 2 g/kg/day 35 days	Oestrus Cycle Regulation Histomorphological Changes	letrozole 1 mg/kg/day of 21 days	Sprague–Dawley Rats (*n* = 24)	The increase in the corpus luteum and antral follicle improved the estrus cycle and reduced the effect of histomorphological changes in developing cystic follicles and ovaries in PCOS.	[7]
2	Kelulut Honey 1 g/kg/day 35 days	Oestrus Cycle, Hormonal Profile Oxidative Stress	letrozole 1 mg/kg/day of 21 days	Sprague–Dawley Rats (*n* = 42)	Co-administration of Kellut honey with metformin or clomiphene contributed positively to the hormonal profile, oxidative stress, and estrus cycle in PCOS rats. Additionally, it had no adverse effects on insulin, blood sugar, and rate of body weight gain in PCOS mice.	[8]
3	Kelulut Honey 1 g/kg/day 35 days	Sex Steroid Receptors	letrozole 1 mg/kg/day of 21 days	Sprague–Dawley Rats (*n* = 42)	This study shows that in PCOS, sex steroid receptors are expressed abnormally and that Kelulut honey treatment can normalize the expression of these receptors.	[9]
4	Kelulut Honey 1 g/kg/day 35 days	Folliculogenesis, Steroidogenic, and Aromatase Enzyme Profile, Ovarian Histomorphology	letrozole 1 mg/kg/day of 21 days	Sprague–Dawley Rats (*n* = 42)	It was found that Kellut honey positively affected Cyp17a1 and Cyp19a1 expression, folliculogenesis, and ovarian histomorphology in PCOS rats, and was more effective when applied together with clomiphene.	[10]
5	Propolis 50 mg/kg and 150 mg/kg	ovarian follicular reserve zona pellucida fibrous tissue	letrozole 1 mg/kg/day of 21 days	Wistar Rat (*n* = 24)	The application of 50 mg/kg propolis had a positive effect on the number of cystic and primary follicles. In the lower concentration groups, however, it did not completely restore PCOS-associated hormones and p53 expression.	[11]
6	Bee pollen 50, 100, and 200 mg/kg	proliferation and apoptosis of granulosa cells	2 mg of estradiol valerate 60 days	Wistar Rats (*n* = 54)	The number of preantral and antral follicles increased in the PCOS group in which bee pollen and metformin were administered together. The number of cystic follicles decreased. TNF-α, NO levels, and Ki67 values were decreased in other groups. Apoptosis increased in bee pollen-applied groups. There was a positive change in PCOS symptoms in both bee pollen and metformin groups.	[12]
7	Bee pollen 50, 100, and 200 mg/kg	Testosterone and Estradiol Levels Apoptotic Markers Total Antioxidant Capacity	2 mg of estradiol valerate 21 days	Wistar Rats (*n* = 54)	Estradiol and testosterone levels and Bcl-2 expression increased in the PCOS group. In the application group, these values decreased. Total antioxidant capacity and expression of Bax, Cas-3, and Sirt1 were decreased, but these values increased in the bee pollen and metformin group.	[13]
8	Royal jelly 100, 200 and 400 mg/kg	Anti-Androgenic Effect	Testosterone 10 mg/kg	Sprague Dawley Rats (*n* = 40)	The T + 200RJ group had higher FSH (Follicule-Stimulating Hormone) levels and lower LH (Luteinizing hormone), testosterone, estradiol levels, lower malondialdehyde, lower glutathione peroxidase activity and higher total antioxidant capacity levels compared to the T group. It also showed recovery in various stages of ovarian follicular development.	[14]
9	Bee venom phonophoresis 1 μg/ml	Case Study		Obese PCOS Women (*n* = 46)	After 7 and 14 weeks of administration, the progesterone value increased but the LH and LH/FSH ratio decreased in the administration group. Dietary bee venom phonophoresis has a positive effect on the treatment of obese women with PCOS.	[15]
10	Bee venom	IL-6, COX-2 and VEGF levels	2 mg of estradiol valerate 60 days	Wistar Rats (*n* = 24)	The theca layer thickness, IL-6 levels, and the number and diameter of cysts were decreased in the HBV group. It showed increased expression of COX-2 and VEGF in the PCOS group. Irregular immunostaining occurred in HBV-treated rats.	[16]
11	Bee venom	Levels of lipids and anti-mullerian hormone	2 mg of estradiol valerate 60 days	Wistar Rats (*n* = 63)	There was a decrease in the corpus luteum diameter in the PCOS group and an increase in the HBV group. Anti-mullerian hormone increased in the PCOS group and decreased in the honeybee venom group. Blood triglycerides and LDL(low-density-lipoprotein) cholestrol levels increased in the PCOS group and decreased in the HBV group.	[17]
12	Bee venom 0.5 mg/kg 14 days	C-reactive protein follicle quality	2 mg of estradiol valerate 60 days	Wistar Rats	Honeybee venom reduced the theca layer thickness, number of cysts, and C-reactive protein levels, and showed the presence of the corpus luteum in the PCOS group. It was thought that honey bee venom would have a positive effect by inhibiting the level of C-reactive protein.	[18]
13	Bee venom 0.5 mg/kg 14 days	Hyperglycemia Hyperandrogenism	Estradiol Valerate (2 mg/100 grB.W)	Wistar Rats	Ovarian weight, testosterone, and estradiol levels increased in the experimental group. These values decreased in the group treated with honeybee venom. The blood glucose levels, the thickness of the theca layer, the number, and the diameter of cysts were reduced in the honeybee venom group compared to the PCOS group. In addition, the corpus luteum was detected in the group in which honey bee venom was administered.	[19]

## Data Availability

Not applicable.
